# The feasibility of using mouthpiece ventilation in the intensive care unit for post-extubation breathing support after acute tetraplegia

**DOI:** 10.1038/s41393-023-00889-z

**Published:** 2023-03-17

**Authors:** Brooke M. Wadsworth, Peter S. Kruger, Craig A. Hukins, Gabrielle A. Modderman, Duncan Brown, Jennifer D. Paratz

**Affiliations:** 1 Physiotherapy Department, Princess Alexandra Hospital, Woolloongabba, QLD Australia; 2 The Hopkins Centre, Menzies Health Institute Queensland, Griffith University, Woolloongabba, QLD Australia; 3 Intensive Care Unit, Princess Alexandra Hospital, Woolloongabba, QLD Australia; 4 Department of Anaesthesiology and Critical Care, The University of Queensland, St Lucia, QLD Australia; 5 Intensive care, Greenslopes Private Hospital, Greenslopes, QLD Australia; 6 Department of Respiratory and Sleep Medicine, Princess Alexandra Hospital, Woolloongabba, QLD Australia; 7 Menzies Health Institute, Griffith University, Griffith, QLD Australia; 8 Burns, Trauma & Critical Care Research Centre, School of Medicine, The University of Queensland, St Lucia, QLD Australia

**Keywords:** Spinal cord diseases, Neuroscience

## Abstract

**Study design:**

A prospective cohort of patients with acute tetraplegia.

**Objectives:**

This study aimed to determine the feasibility of using mouthpiece ventilation (MPV) in the intensive care unit (ICU) for patients who are extubated after suffering an acute cervical spinal cord injury (CSCI).

**Setting:**

ICU, Princess Alexandra Hospital, Brisbane Australia.

**Methods:**

New admissions to ICU in the 14 months between April 2017 and June 2018 with a CSCI who underwent intubation were assessed for inclusion. MPV was provided to consenting participants (who were deemed likely to be able to maintain ventilation on their own) at the time of extubation and was utilised in addition to standard care while participants were awake. MPV settings, usage, and support hours to educate and facilitate MPV were collected. Feedback from participants and clinical staff was gathered throughout the study. Pre- and post-extubation measures of forced vital capacity (FVC), the frequency of endotracheal suction of sputum, and gas exchange using ventilation-perfusion ratios were recorded along with the incidence of reintubation.

**Results:**

Fourteen participated in utilising MPV with 16 episodes of extubation. The average time per participant to have MPV titrated and bedside data collected was 178 minutes. Data from 16 episodes of extubation have been included. Three of the 14 participants failed initial extubation. Feedback from participants and clinicians has been positive and constructive, enabling MPV settings to be adapted to the person with acute CSCI during this pilot study.

**Conclusion:**

MPV is feasible to use post-extubation for people with CSCI in ICU. Pressure control mode MPV was deemed the most suitable for newly extubated acute CSCI patients. Intensive clinical support is required initially to provide education prior to MPV, and at the time of extubation for both patient and treating clinicians. Both report it to be a useful adjunct to ICU treatment.

## Introduction

One in five patients with acute cervical spinal cord injury (CSCI) fails extubation [1], with the odds almost three times greater in those with complete CSCI [1]. It is well recognised that excessive tracheobronchial secretions at the time of extubation, together with a weak or ineffective cough can lead to impaired airway competency and, consequently, to extubation failure [2]. Once the patient is deemed medically stable and unlikely to be dependent on long-term mechanical ventilator support (as might be expected with complete injury at C1-C3), the decision must be made whether to extubate or progress directly to a tracheostomy. This decision is usually made in the intensive care unit (ICU), where staff understand how acute SCI can uniquely affect respiratory muscles and breathing mechanics.

Post-extubation treatment in our centre involves the delivery of high-flow oxygen and regular early and intensive physiotherapy [3]. Non-invasive ventilation (NIV) for additional support of the respiratory system is also considered. It may be used as part of standard care post-extubation or be offered when the patient has marginal ventilation and oxygenation. This therapy is associated with disadvantages including interface leaks, mask intolerance, drying of airways and secretions, risk of skin pressure areas and the impact of a facial mask on speech and oral intake [4]. Mouthpiece ventilation (MPV) is a more recent revived technique for the delivery of ventilatory support which has been validated in other neuromuscular conditions [5, 6], both with stable patients in the community [7–9], and during acute respiratory exacerbations [10, 11]. This therapy involves the on-demand delivery of ventilatory support through a mouthpiece without the requirement for a facial mask allowing the person to determine the frequency and timing of ventilatory support. Bach and colleagues [12–14] have shown that MPV is an option for acute CSCI in their hospital, but this has not been validated in other centres. Therefore, the use of prophylactic on-demand MPV which can be triggered by the user is novel and warrants consideration in the acute care environment for those patients with CSCI.

This study aimed to assess the feasibility of MPV use in the ICU for patients who were extubated after suffering an acute CSCI. The aims of this study were to determine the nature and the amount of education and assistance (both staff and participants) required to adequately implement MPV; determine participants and clinicians’ experience using MPV; determine if any baseline characteristics were likely to influence the use of MPV and identify any barriers and enablers from a practice perspective.

## Methods

### Participant selection and baseline demographics

All patients who were admitted to the Princess Alexandra Hospital ICU between April 2017 and June 2018 with cervical spinal cord trauma requiring intubation and were being considered for extubation, were assessed for study inclusion. Patients were excluded if pregnant, under the age of 18 years, had experienced a head injury that prevented them following commands or had no demonstrated neurological impairment from their injury. Once the medical team determined the patient was ready for extubation the patient was approached to participate in the study and provide informed consent.

Post extubation usual care was carried out which included use of manual assisted cough and a mechanical cough assist machine for airway clearance. Airway clearance was conducted by physiotherapy and nursing staff at the time of extubation and then regularly post-extubation. This was combined with position changes and other physiotherapy techniques to maintain airway clearance and optimise ventilation. High-flow oxygen via a nasal cannula (HFNC) was utilised to assist with oxygenation and provide some gentle positive pressure using a flow of between 10 to 50 litres per minute as part of usual care.

### MPV intervention

**Fig. 1:**
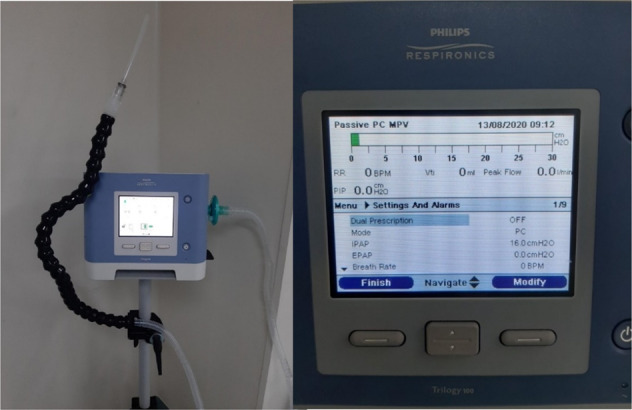
Philips Trilogy 100 with tubing, straw interface and setting screen.

The MPV intervention was provided in addition to usual post-extubation treatment. MPV was delivered by the Philips Trilogy 100 portable ventilator (Murrysville, Pennsylvania, USA). The MPV feature of this ventilator incorporates a “kiss” trigger with signal flow technology which detects when a user engages and disengages from the mouthpiece to deliver on-demand ventilation. The adjustable arm housing the circuit of tubing and interface is shown in Fig. 1. The adjustable arm allows the mouth interface to be positioned at any angle the user feels most comfortable to access it. The mouthpiece connection was either a 15 mm angled mouthpiece or a vinyl or polyethylene straw tubing and this was determined by participant preference for comfort. MPV was available at the time of extubation to deliver breaths so the participant could take a supported breath at any time when they felt fatigued, when they wished to talk, when they sat up in or out of bed, and for airway clearance assistance.

Initial settings for MPV were determined depending on oxygen saturation, FVC and set up by the study team (BW, GM, PK, CH) and approved by the treating medical ICU specialist. The mode of MPV for inspiratory support was set to pressure control mode (PC MPV) to allow for leak compensation delivered through an open, single-lumen tube. Titration of the inspiratory pressure over the course of the study was carried out by investigators BW, GM, or CH. The patient was acclimatised to MPV using a starting inspiratory pressure of 10 cm H_2_O, inspiratory time of 1 second, inspiratory rise of 1 which was progressively titrated according to patient comfort and clinician assessment of the adequacy of ventilation based on chest and abdominal wall motion, auscultatory findings and measurement of oxygen saturation. Parameters, including the inspiratory time and rise time were adjusted to facilitate suitable breath delivery dependent on the patient’s spontaneous respiratory rate and breathing pattern. Participants were able to control the frequency of augmented breaths by choosing when to utilise the mouthpiece. The inspiratory support was also adjusted based on feedback on the amount of inspiratory pressure and the participants ability to adjust to the duration of the ventilatory support (how long they remained on the mouthpiece each breath).

The device was set-up to only deliver inspiratory positive airway pressure (IPAP) support with no expiratory positive airway pressure (EPAP) with exhalation occurring passively. Oxygen and humidification were delivered via high-flow nasal cannula (HFNC) as directed by the treating medical team and not influenced by this study. Physiotherapy and airway clearance interventions were conducted as per usual care. The treating medical specialist evaluated clinical progress of the patient post extubation independent of the study and determined the need for additional respiratory therapies or reintubation without any influence from the MPV research team. The MPV intervention was provided from the time of extubation until the participant was either discharged from ICU, no longer utilized MPV or required reintubation.

Prior to recruitment commencement, the study team had completed eight hours overall of education to nursing, medical, and physiotherapy ICU staff which covered the mechanics of breathing in CSCI, how MPV works, and the details related to the study aim, procedures, and outcomes.

### Primary outcomes

The primary outcome measure was the feasibility of the MPV intervention considering a combination of its tolerability, acceptability, and implementation time. The time taken to complete set-up of the device, educate staff to support each participant and to use the equipment, adjust equipment settings/interface and record bedside outcomes was recorded for each participant. The participant’s comfort with the interface and interface preference was noted throughout. Participant adherence to MPV was determined by device download using Direct View software. The number of augmented breaths was calculated from recorded breathing frequency data. The participant’s experience of MPV use were recorded directly throughout the study and then a semi-structured interview was conducted at the study conclusion. Nursing, medical, and physiotherapy staff working with each participant during this study and MPV use were invited to complete a feedback survey (Appendix 1).

### Secondary outcomes

Measures of forced vital capacity (FVC) using a Wright respirometer (Bird Healthcare, VIC, Australia) were collected at the same time of day from immediately prior to extubation connected to the endotracheal tube (ETT), within 30 minutes of extubation (via the mouth), and then daily until participants were either discharged from ICU or required re-intubation. Measures of FVC were taken with the bed head raised to 10 degrees and then again at 45 degrees as per the bed inclinometer without an abdominal binder in place. The best of three attempts at FVC was recorded with nose occlusion provided by the investigator. The modified BORG scale [15] was used to assess the participants self-report of breathlessness to assist with acknowledging their current respiratory state and evaluating setting changes. FVC and Borg scale were included to allow repeatable measures of clinical breathing ability and guide setting adjustment. BORG was used in pre and post setting adjustment to determine if the increase in MPV pressure or inspiratory time eased any feeling of perceived breathlessness. Whilst tidal volume was not collected as part of the outcomes measured, a target of 10cc/kg ideal body weight was initially utilized on extubation which then progressed to 20–25 cc/kg. Not all this pressure and volume of air would be delivered as a tidal volume as this is an open system, participants could leak excess air through their mouth or nose if they wished to not take in the full amount.

Frequency of endotracheal suction data was taken from nursing hourly observation records at the study conclusion. The indications for invasive airway suction were not established between clinicians and the research team prior to the study commencement. This was based on usual clinical practice. Gas exchange as measured by ventilation/perfusion ratios (PaO_2_/FiO_2_) was collected with the lowest PaO_2_/FiO_2_(P/F) value in the 24 hours prior to extubation, and at the time of extubation being recorded. Measures of pre-extubation FVC, PaO_2_/FiO_2_, and endotracheal suction data were included as per Berney et al [16] to ascertain if these measures provided any airway management predictive function in our cohort.

Study data were collected and managed using REDCap electronic data capture tools [17]. Results were summarized as percentages of responses, mean (s.d) or median (IQR) as appropriate.

### Reporting summary

Further information on research design is available in the Nature Portfolio Reporting Summary linked to this article.

## Results

All those using MPV reported it easy to use. Two-thirds of the participants used a combination of the straw and mouthpiece connection for the MPV. Ninety-two percent of patient participants felt that MPV improved their sputum clearance and their breathing.

**Fig. 2:**
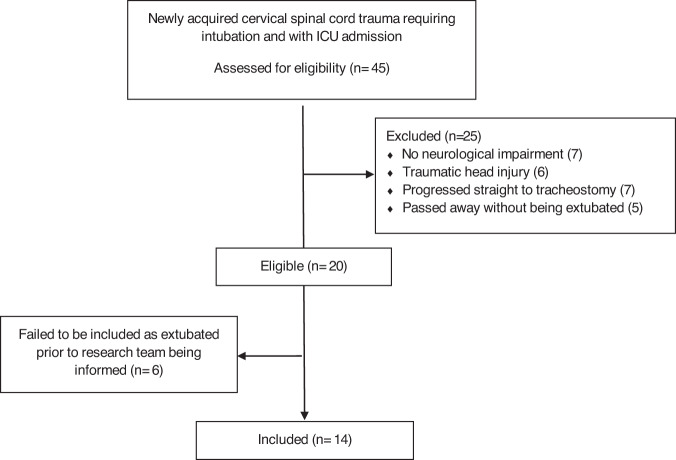
Consort Diagram.

Fourteen participants with a new CSCI were included and consented to use MPV (see Fig. 2. Consort Diagram).

Their baseline characteristics are reported in Table 1. No participants withdrew from the study. Data from the 16 episodes of extubation across the 14 participants (three participants failed initial extubation with two going on to be extubated again and use MPV) is reported in Table 1.

**Table 1:** Demographics and baseline characteristics.

Participant #	Age	Sex	Cause of SCI	ICU ISNCSCI	Pulmonary condition before SCI	Smoking history	Reason intubated	Injury to Surgery (hours)	Length of Intubation (hours)	Injury to extubation (hours)	ICU LOS (days)	APACHE II	APACHE III	FVC supine (millitres) Pre-extubation	Sputum- frequency of suction at ETT	Pa02/Fi02: Lowest in previous 24 hours	Pa02/Fi02 immediately pre-extubation	Clinically defined pneumonia at time of extubation	ICU LOS (days)	MPV day one pressure support (cmH2O)	MPV final pressure support (cmH2O)	MPV titration total time (minutes)
1	34	M	Diving	C4 B	None	Past	Resp	19.92	136	140	13	10	28	0.70	<hourly suction	227	300	Yes	13	12	15	210
2	42	M	Fall from height	C3 C	None	Current	Surg	52.87	68	120	9	8	22	1.50	<hourly suction	410	410	No	9	22	22	240
3	72	M	Diving	C3 A	Right sided pleural effusion of unknown cause requiring pleurodesis 1 month prior to accident	Past	Resp	51.52	27	77	9	11	40	1.00	<hourly suction	140	310	Yes	9	10	14	70
4	45	F	Fall from height	C5 B	None	Past	Surg	12.57	21	32	4	11	35	2.21	<hourly suction	300	300	No	4	16	18	90
5	27	F	MVA	C4 C	None	Never	Surg	10.73	30	40	4	17	46	0.85	<hourly suction	453	453	No	4	10	10	70
6	21	M	Diving	C6 A	None	Past	Resp	9.55	196	221	14	22	72	1.20	<hourly suction	236	363	No	14	10	15	170
7^C^	21	M	Diving/Drowning	C5 B	None	Current	Resp	41.7	348, 90^a^	348	22	14	40	2.00, 2.05^a^	<hourly suction	170, 260^a^	189	Yes	22	18	14	200, 110^a^
8	62	M	MBA	C4 D	Asthma - recent antibiotics for chest infection	Never	Surg	19.68	17	35	2	12	37	2.63	<hourly suction	254	335	No	2	18	18	100
9	64	M	Diving	C4 C	None	Never	Resp	41.63	210	210	10	18	51	1.19	<hourly suction	164	194	Yes	10	10	14	70
10^c^	62	M	Diving/Drowning	C4 B	None	Never	Surg	57.98	38, 120^a^	96	12,7^b^	16	54	1.95, 1.92^a^	twice hourly suction	217, 240^a^	307	Yes	12,7^b^	20	22	185, 225^a^
11	24	F	Diving	C4 A	None	Never	Surg	30.25	22	46	6,13^b^	8	40	1.55	<hourly suction	290	290	No	6,13^b^	12	12	300
12^d^	32	M	MBA	C5 A	None	Current	Surg	44.17	131	138	17	4	25	Not collected	<hourly suction	293	293	No	17	UTU	UTU	70
13	56	F	Fall from height	C6 B	OSA	Current	Surg	14.33	38	52	10	16	46	Not collected	hourly suction	234	303	No	10	14	18	310
14	25	M	MBA	C8 B	None	Never	Surg	6.42	34	38	4	8	23	1.10	<hourly suction	436	440	No	4	10	10	70

^a^second intubation.

^b^second ICU stay.

^c^failed initial extubation, 2nd extubation successful.

^d^failed initial extubation progressed to tracheostomy.

*MVA* Motor vehicle accident.

*MBA* Motor bike accident.

*OSA* obstructive sleep apnea.

*Resp* Respiratory.

*Surg* Surgery.

*UTU* unable to use.

Just over two hundred clinical staff received education prior to this study starting during designated group education timetabling. Almost all clinical staff had no prior knowledge of MPV but 68% thought it would likely help the patient a lot. The time involved in setting up and establishing MPV at the time of extubation ranged from 70 minutes–100 minutes during the initial post-extubation contact. All participants required a follow-up review of settings and use within 1–2 hours post-extubation which lasted from 5 minutes to 20 minutes. The overall bedside time spent titrating MPV, measuring FVC, and collecting participant and clinician feedback for the 14 study participants was 2490 minutes (41.5 hours) over 14 months. The range of time per patient spent was from 70 minutes to 410 minutes with a median of 135 (IQR 230).

The time spent with MPV set-up and titration was dependent on participant alertness, comfort levels in relation to pain, breathlessness, and ability to connect with the mouthpiece. The four participants requiring over 300 minutes of MPV titration included the two who went on to utilise MPV at second extubation, one who had obstructive sleep apnoea, and the other required readmission to ICU due to untreated pneumonia.

Feedback from clinicians was positive and constructive with requests for ongoing, one-to-one education at the bedside as part of change of shift handover as the study progressed. Eleven percent of staff participating in the study felt that there was not enough education provided on MPV prior to, or as part of the study (although 89% felt there was enough education). Patient participants reported they felt it helped when they wanted a louder voice and when they were feeling fatigue, but it was annoying if the interface was left positioned too close to their face or eyes. Care was taken to minimize any experience of dizziness by using a lower pressure and fewer number of breaths initially as the participant became familiar with it on extubation.

**Fig. 3:**
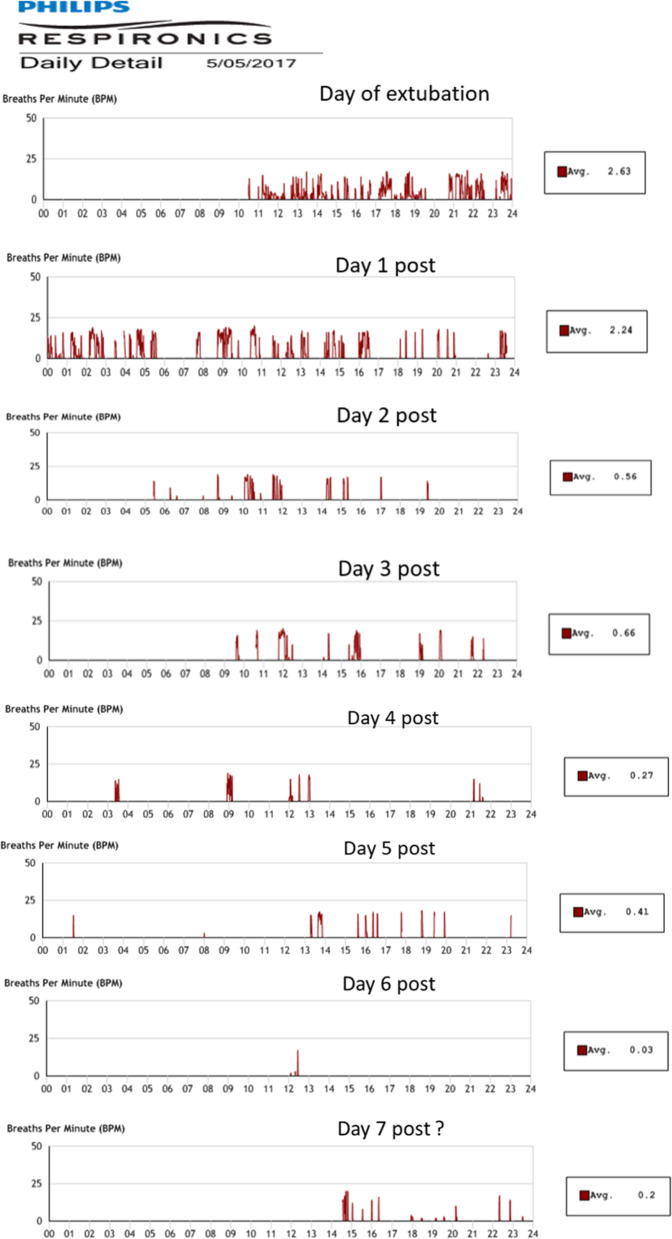
Participant 1 data showing MPV use over 24 hour period to indicate engagement.

An example of the frequency of MPV use during 24-hour periods is shown in Fig. 3 (Participant 1 data shown). It indicated frequent use during the first 30 hours after extubation just after 10am, then intermittent use during the following days. There was an increase in use on day 7 as the participant was transferred to an orthopaedic ward and requested ongoing use. The IPAP pressures used is shown in Table 1.

The preferred MPV interface on initial extubation was the polyethylene straw tubing, which was cut to size, and angle adjusted to be easily engaged with by the participant. Once the participant became familiar with MPV and approaching ICU discharge, the angled firmer mouthpiece was more commonly utilized with particular care taken with those participants who did not have a full set of teeth.

Three participants failed to engage effectively with the MPV due to fatigue and altered consciousness level/delirium associated with respiratory failure and were subsequently reintubated within 72 hours of extubation. Two of these participants had experienced near-drowning accidents, being revived at the accident scene. The other participant experienced multi-trauma to the ribs, lungs, thorax and found to have a TBI. All suffered rib fractures reducing positioning options and manual therapy techniques for airway clearance. There appeared to be no other similarities when considering ISNCSCI, airway or smoking history or intubation reason or time.

Pre-extubation FVC was collected for 14 of the 16 episodes of extubation. Two were missed as one participant was extubated just prior to the research team being available at the bedside and the other due to excessive ETT cuff leak resulting in need for extubation and then the research team were informed. The mean pre-extubation FVC across the 14 extubations was 1.49 litres (range 0.7–2.63 litres). Endotracheal suction frequency and P/F ratios prior to extubation were recorded and presented in Table 1. Two participants (participants 1 and 5) had a pre-extubation FVC of less than a litre but were able to utilise MPV and remained extubated. One participant (participant 10) had required twice hourly endotracheal suction prior to extubation and was able to use MPV initially but then required reintubation. The one participant who had a tracheostomy after failing extubation, did not have a pre-extubation FVC measured and was not able to engage with MPV for more than a few hours post extubation due to delirium and respiratory failure.

## Discussion

Our study has shown that using MPV in the ICU for post-extubation in people with CSCI is feasible. While it requires additional clinical support initially, we have shown that it is well accepted by the patients and the clinical team and can offer an alternative or complimentary therapy to other forms of NIV delivered by a facemask or nasal interface.

Feedback from participants allowed MPV to be adjusted to the individual with mouth interface options changed to suit comfort and ease of positioning. The level of positive pressure delivered was titrated to initially support inspiratory effort by easing the work of breathing and then focusing on augmenting a deeper breath when the participant felt the need for more air. This adaptation process is likely to have assisted with giving the patient as sense of control over their breathing and provided the clinical team with another option to enhance breathing support. Whilst clinician feedback about MPV was favourable, the need for further one to one education and support for this novel treatment is required to fully evaluate its optimal delivery.

With little published with respect to effective prescription of MPV settings for various patient populations our study has presented new information on MPV in patients with acute spinal cord injury being recently extubated. The settings required for a patient with a chronic condition such as Duchene Muscular Dystrophy in the community is very different to those required for acute traumatic CSCI in the Intensive Care Unit. The patient with a neuromuscular disease (NMD) has usually used nocturnal NIV prior to being introduced to daytime NIV via mouthpiece interface, therefore familiarity and ability to give useful feedback on settings and interfaces such a straw or mouthpiece is quite different to the acutely injured first time user. We used pressure control MPV in this study which allows for leak compensation and the more predictable delivery of inspiratory pressures. Assist control volume mode (AC) mode is often advised in other conditions to facilitate a breath/air stacking manoeuvre during awake hours, performed by teaching the patient to stack consecutive volumes of air delivered from the ventilator until the lungs are maximally expanded which is helpful for maintaining airway clearance [8, 14, 18, 19]. However, AC mode is not preferred in our centre with patients with acute CSCI as they could not coordinate the breath stacking benefit. Our team acknowledges that patients with chronic CSCI have been able to utilise breath stacking in AC mode to augment a deep breath with MPV delivered by the Philips Trilogy ventilator. Whether the changes to chest and abdominal wall compliance and the resolution of spinal shock impact on the ability to use different MPV modes is worthy of further consideration.

We found it useful to introduce MPV by supporting the short rapid breaths with short inspiratory and rise times at low pressure (i.e., 10 cm H_2_O initially). As the patients’ breathing improves the principles for adjusting the MPV settings include a shift towards longer, deeper, and higher-pressure inspiratory breaths (i.e., 18–22 cm H_2_O) which the patient uses less frequently. The time taken for this shift to occur varied in this study from several hours post-extubation to several days depending on the participants’ ability to breathe for themselves, the extent of medical complications, and airway clearance frequency. By this point, the participant was usually using MPV to supplement airway clearance, voice volume, and “getting their breath back” after bed baths or procedures that required physical effort. The participants self-reported level of breathlessness using the Borg scale assisted with assessing setting changes and breathing comfort throughout the study. If the “kiss trigger” feature of the Philips Trilogy 100 ventilator bothered the patient, we found applying a filter at the user end to reduce background flow was helpful.

Of the three participants who did not effectively engage with MPV and were reintubated (one to have a tracheostomy and two who then utilised MPV effectively on second extubation), it is useful to consider the influence of known factors that influence decision-making, such Berney’s et. al [16] classification and regression tree (CART) model. In this model, from a single centre, factors such as patients with an FVC of less than 830 ml (11.9 ml kg−1), abundant pulmonary secretions (suction >hourly) and poor gas exchange (p/f ≤ 188.8), were predictive of airway management issues for patients having acute CSCI. Although two of the three participants who failed extubation (third one did not have FVC pre-extubation measures done) had clinically adequate FVC measures, both had clinically defined pneumonia at time of extubation, one had twice hourly suction requirements and the other had impaired gas exchange with a P/F of 170. We did see one of our participants with an FVC of less than 830mls and a successful extubation with support of MPV. However, with only 16 occasions of extubation included in our study the CART continues to require further validation to determine its usefulness for clinicians as an extubation clinical decision-making tool and the impact MPV may have on modifying this process requires further evaluation.

Acute cognitive impairment was a common clinical feature associated with treatment failure. Three participants with altered levels of consciousness required re-intubation. This is not surprising as the participant needs to be cooperative and become acclimatised to therapy when first introduced and should be actively involved in the adjustment of settings which is not possible when cognitively impaired.

Whilst this study sought to determine whether MPV was feasible for people with CSCI in ICU, it recognised but did not record, the impact of other variables on the patients post extubation journey. The patient’s level of rest and sleep quality in the days preceding extubation needs to be acknowledged. In addition, the influence of positioning on extubation success has not previously been evaluated. The unique consideration for motor complete CSCI patients with non-functioning abdominal muscles is that FVC is greater in supine than sitting within the first 12 months of injury [20]. This contrasts with healthy individuals in which FVC is greater in the seated posture. For this reason, it should be noted that positioning impacts post-extubation breathing ability and adds to the variables needing to be considered when evaluating any respiratory intervention for this cohort. Additionally, the experience of intubating the patient and whether it was considered a difficult airway intubation would help understand airway failure risks and to plan early if extubation failure is impending. Reporting on extubation treatments utilised such as intensive physiotherapy service, airway clearance options, use of other forms of NIV within ICU will help further understand different ICU’s threshold to re-intubate or tracheostomise. Unfortunately, we were unable to record specific intensity of MPV delivered during this study as the device only provided an indicative value. As technology and competition continues in the field of NIV devices it is likely that compliance and dosage data will become more robust.

In Table 1, the “MPV day one pressure support” ranged from 10–22 cmH2O (Mean 14, Inner Quartile Range 10,18) and the “MPV final pressure support” ranged from 10–22 (Mean 15.5, Inner Quartile Range 14,18). We commenced on a pressure support of 10 cmH2O and then titrated the pressure support in increments of 2–4 cmH2O as tolerated. Daily, the study team attempted to increase the pressure support. It is notable that the 25% percentile increased from 10 to 14 cmH2O over the course of the titration. The data suggest that patients generally tolerate progressively increasing pressure support over time. It is further notable that the 75% percentile did not increase which may suggest a pressure support of 18 cmH2O as a reasonable target goal for the first application of MPV (NB: this is not a ceiling if patients can tolerate more pressure). Ultimately, the highest achieved MPV pressure support of 22 cmH2O suggests a practical ceiling beyond which there may be diminishing returns based on the limits of patient pressure tolerance.

In our clinical experience, it is common for the acute CSCI patient upon extubation to be anxious with a temporarily higher respiratory rate, a fear of not being able to get enough breath in, and occasionally a sore throat or dysphagia. The ability to take a deep breath on demand with MPV was the most common positive feedback received from participants. This study provides the first clinical evidence to guide use and settings for MPV in people with acute SCI. Whether MPV is an effective post-extubation treatment option to reduce pulmonary complications, ICU LOS or prevent extubation failure, needs to be determined in a larger study.

## Supplementary information

Clinician Participant MPV Questionnaire

Reporting Summary

## Data Availability

The datasets generated and/or analysed during the current study are available from the corresponding author.
