# Supplementary material for: The feasibility of using mouthpiece ventilation in the intensive care unit for post-extubation breathing support after acute tetraplegia
Source: Spinal Cord. 2023 Mar 17;61(6):330–7. doi: 10.1038/s41393-023-00889-z (PMC10328823; doi:10.1038/s41393-023-00889-z)
Supplement: Supplementary file 1 — Clinician Participant MPV Questionnaire [file 41393_2023_889_MOESM1_ESM.docx]

**Clinician Participant MPV Questionnaire**

The following questions are related to the mouthpiece ventilation (MPV) machine you used recently as part of study **The Feasibility of Using Mouthpiece Ventilation Post Extubation for Acute Tetraplegia in the Intensive Care Unit**. Please circle only one response for each statement. Please be sure to choose a response for all statements.

1. **Prior to the commencement of this study, your level of knowledge about MPV, indications for use and how a device could deliver MPV was;**

| Not knowledgeable about | Somewhat knowledgeable about | Knowledgeable about | Very knowledgeable about |
| --- | --- | --- | --- |

1. **After initial education about this study titled; Mouthpiece ventilation post extubation for acute tetraplegia, to what extent did you believe it would assist the participants breathing?**

| Make no difference | Somewhat of an improvement | Likely help a lot |
| --- | --- | --- |

1. **The research team provided me with enough education, demonstration and direct support to allow the participant to utilize MPV once the research team had set-up**

| Strongly disagree | Disagree | No opinion | Agree | Strongly agree |
| --- | --- | --- | --- | --- |

1. **Did you feel there were any negative impacts of the MPV and if so what were they?**
2. **Did you feel there were any positive impacts of the MPV and if so what were they?**

**Please turn over for final question………………………………………………………………………………………………**

1. **Any comments/suggestions about MPV or future study considerations using MPV?** (e.g. education support provided, adjustment frequency, ability to access machine/mouthpiece, alarms, if a different breathing support used overnight):

Thank you for your comments and time supporting this feasibility study.

Brooke Wadsworth (Chief Investigator, Physiotherapist)
